# Social, economic and family factors associated with binge drinking in Spanish adolescents

**DOI:** 10.1186/s12889-020-08605-9

**Published:** 2020-04-17

**Authors:** Ana Magdalena Vargas-Martínez, Marta Trapero-Bertran, Toni Mora, Marta Lima-Serrano

**Affiliations:** 1 Department of Nursing. Faculty of Nursing, Physiotherapy and Podiatry, Universidad de Sevilla, Seville, Spain; 2 Faculty of Economics and Social Sciences, Universitat Internacional de Catalunya, Barcelona, Spain; 3 Research Institute for Evaluation and Public Policies (IRAPP), Universitat Internacional de Catalunya, Barcelona, Spain

**Keywords:** Binge drinking, Adolescence, Socioeconomic factors, Intervention

## Abstract

**Background:**

The main aim of this study was to determine the socioeconomic and family factors associated with binge drinking (BD) in Spanish adolescents who participated in a web-based computer intervention for the prevention of binge drinking known as *Alerta Alcohol*.

**Methods:**

Longitudinal analyses were carried out in a sample of Andalusian adolescents aged 15 to 19 enrolled in public schools, which was part of a two-arm cluster randomized controlled trial with an intervention group (IG) who received the *Alerta Alcohol* programme and a control group (CG) who did not receive any active intervention. Panel count data and the following econometric procedures were used: negative binomial, a two-part model and a finite mixture model. The endogenous variable in all models was the number of BD occasions in the last 30 days. A total of 1247 subjects in the pre-intervention period, with an average age of 16.8 years, plus 612 adolescents in the follow-up period (4 months later), were included in the analysis.

**Results:**

In relation to findings, being older (*≥* 17 years old), having more pocket money and higher family alcohol consumption were associated with greater BD. By contrast, subjects who completed the questionnaire on Wednesday, Thursday or Friday, further from the previous weekend, indicated a lower number of BD occasions.

**Conclusions:**

Our results suggest the need to include families, especially parents and siblings, in interventions aimed at preventing alcohol use among adolescents, given the association shown between BD and both family alcohol consumption and weekly pocket money or availability of money to adolescents. Given the findings with regard to age, future research aimed at intervening in early adolescence to prevent BD would be justified.

**Trial registration:**

(ClinicalTrials.gov): NCT03288896. Registration date: September 20, 2017. “Retrospectively registered”.

## Background

According the World Health Organization (WHO), alcohol was the seventh cause of disease and premature death among the world’s population in 2016. Also, alcohol is the cause of more than 200 health conditions, in addition to diseases with a high mortality burden such as liver cirrhosis, cancer and cardiovascular diseases [1, 2]. In Europe, alcohol accounts for 10.1% of all deaths and 10.8% of all disability-adjusted life years (DALYs) [1, 2].

With regard to alcohol use in different life stages, it is known that alcohol abuse is a public health concern across all age groups. Nevertheless, it is important to highlight that alcohol is the most widely used substance among adolescents in both Europe and North America [3]. In Spain, the alcohol-related disease burden for the young population aged 15 to 29 years is estimated at the equivalent of 786,479 DALYs (16% of the total for the Spanish population) [4]. Specifically, for young people, alcohol has numerous consequences, mainly on morbidity, such as the effects on the brain [5], visuospatial memory deficits, and attention impairments [6]; alcohol use can also increase the risk of unprotected sex or of involvement in an aggressive incident or an activity with a high risk of injury [7].

As is known, the pattern of alcohol consumption differs according to the age of the population. It has been pointed out that binge drinking (BD) among young people is a particularly prevalent pattern of alcohol consumption, characterized by the intake of high amounts of alcohol (at least five standard drinks for men and four standard drinks for women) on a single occasion [8–11]. The definition of BD is controversial, and multiple definitions exist [12, 13]. Additionally, there are different definitions of “standard drink unit”, as the amount that constitutes one unit of alcohol in grams of pure alcohol differs by country [13].

In Europe, the European School Survey Project on Alcohol and Drugs (ESPAD) estimated that about 35% of adolescents aged 15–16 years had engaged in at least one BD occasion in the past 30 days in 2015 [14]. The latest survey on drug use in secondary education in Spain, known as ESTUDES, reported that the prevalence of BD in the past month among students aged 14 to 18 was 31.7% (approximately one in three adolescents) in 2016. The highest rate of BD occurs between the ages of 17 and 18, reaching up to 58.9% among boys and 54% among girls [15].

In addition to the acute consequences associated with alcohol consumption mentioned above, Bockërman et al. [16] found that BD, in particular, is negatively associated with employment months and therefore also with subsequent long-term adverse labour market outcomes. Furthermore, BD is associated with three of the main causes of death among young people, particularly among female adolescents: unintentional injury, homicide, and suicide [17, 18]. For that reason, it is necessary to know the determinants that lead adolescents to acquire these behaviours. The effects of certain social, economic and family factors on health-related behaviours can influence disease outcomes, manifesting themselves in later stages of life [19].

In the current literature regarding the factors associated with this alcohol consumption pattern, the study carried out by Jander et al. [20], through focus group interviews conducted with adolescents aged 16 to 18, found some factors that could influence propensity to engage in binge drinking, such as parental attitudes, being at a party or in a bar with friends on weekend days, peer influence, family affluence and policies concerning the availability of alcohol in the environment. One of the factors that has been shown to have an association with binge drinking is socioeconomic status, as measured by the mother’s educational level, the parents’ occupational status and the subjective perception of wealth [21]. However, the majority of studies analysed in a systematic review carried out by Kwok and Yuan [22] found no relationship between parental socioeconomic status and binge drinking in adolescents. This finding is mentioned due to the variation in measurements of binge drinking and parental socioeconomic status.

Educational aspirations of young people have also been associated with alcohol use. Liu et al. [23], in a study that looked at 15-year-old adolescents from the Finnish Health Behaviour in School-aged Children study from 1990 to 2014, noted that both boys and girls with low educational aspirations were more likely to report frequent drunkenness than those with high educational aspirations.

Regarding family factors, it has been found that the children of parents who engage in bouts of heavy drinking are more likely than children of abstainers to have heavy drinking occasions [24]. They are also more likely to have higher household income, suffer early-life stress and traumatic events, have a negative relationship with their fathers during childhood and adolescence, live in an environment where alcohol is easily accessible and begin using alcohol at an early age [25]. However, there is also literature that did not find a significant association between parental drinking and the different patterns of alcohol use in adolescence in terms of both quantity and frequency of alcohol use [26].

Although some studies analyse the association between different alcohol consumption patterns in adolescents, there is scarce updated knowledge about the influence of socioeconomic factors on binge drinking, which are needed to plan and implement strategies to prevent this particular and frequent pattern of alcohol use [27]. Moreover, Kendler et al. [28] have suggested that, given the complexity of the relationship between socioeconomic variables and alcohol-related behaviours during adolescence there is a need for future studies using longitudinal data for establishing causal relationships.

Hence, the main aim of this work was to explore the social, economic and family factors associated specifically with binge drinking among Spanish adolescents between 15 and 19 years old.

## Methods

### Design and sample

Data from a longitudinal study, a two-arm cluster randomized controlled trial with an intervention group (IG) and a control group (CG) randomized at the school level, for prevention of BD in adolescents known as *Alerta Alcohol* were used in this work *(more information about the design of the study can be obtained from the published study protocol; see Lima-Serrano* et al.*, 2018)* [29]. *Alerta Alcohol* is a web-based computer-tailored programme consisting of six sessions, of which sessions 1 and 6 consist of assessment questionnaires and sessions 2 to 5 are aimed at providing feedback through preventive messages and personalized information to intervene on alcohol use. Results in the IG were compared with the absence of intervention. Hence, the CG only received sessions 1 and 6, whereas the IG participated in all the sessions.

A sample was selected from the overall population of Andalusian adolescents aged 15 to 19 years enrolled in public higher secondary schools, lower secondary schools and lower vocational training schools. Specifically, to be included, adolescents had to be enrolled in the fourth course of compulsory secondary education, the first course of higher secondary education or the first course of vocational training (equivalent to 10th and 11th grades in the United States of America). This sample was randomized at school level. For the recruitment of schools, collaboration was requested from the Educational Plans and Programs Service of the Department of Education, Culture and Sports of the Government of Andalusia. Schools were contacted by various means (telephone, email and through visits). A total of 16 schools were randomized into the control group or the intervention group. Of the schools randomized to the control group, one withdrew its participation before the baseline period.

The average number of adolescents per school was around 83 students. The school councils and the youths’ parents were informed in advance of the study’s objectives and methods. The sample was calculated using the online GRANMO tool, taking into account the prevalence of adolescent binge drinking in Spain (33.1%) [15] and estimating that the intervention would reduce consumption by 10%, accepting a *p* value of < 0.05 and a statistical power of 0.80, for a two-sided test. Based on the study by Jander et al. [30], a dropout rate of about 50% was anticipated. The arc sine approximation was used.

The sample comprised 1247 subjects in the baseline period (January–February 2017) and 612 (49.07%) subjects in the follow-up period (April–May 2017) (see Table 1).

**Table 1:** Characteristics of subjects at baseline period (*n* = 1247) and at 4-month follow-up (*n* = 612) by intervention and control group

	Intervention group		Control group	
Description of variable	Baseline Mean (SD)	Follow-up Mean (SD)	Baseline^**a**^ Mean (SD)	Follow-up^**b**^ Mean (SD)
*Socioeconomic*				
Age at the beginning of programme^1^	16.866 (1.06)		16.681 (1.04)***	
Family functionality: APGAR^1^	1.677 (0.57)		1.727 (0.53)	
Mother’s schooling years^1^	11.211 (3.33)		11.807 (3.28)**	
Father’s schooling years^1^	11.040 (3.37)		11.357 (3.58)	
Pocket money (weekly)^1^	10.610 (9.08)		11.206 (9.61)	
Female^2^	0.535 (0.50)		0.523 (0.50)	
Spanish^2^	0.954 (0.21)		0.936 (0.24)	
Catholic^2^	0.623 (0.49)		0.604 (0.49)	
No religion^2^	0.317 (0.47)		0.337 (0.47)	
Type of family composition: nuclear^2^	0.741 (0.44)		0.743 (0.44)	
Current job situation of the mother^2^	0.634 (0.48)		0.691 (0.46)	
Current job situation of the father^2^	0.798 (0.40)		0.727 (0.45)**	
Good economic situation at home^2^	0.449 (0.50)		0.491 (0.50)	
Economic difficulties at home^2^	0.364 (0.48)		0.291 (0.46)**	
*Alcohol consumption*				
Number of BD occasions^1^	1.125 (1.90)	0.876 (1.74)	1.081 (1.91)	1.065 (2.15)
Frequency of alcohol use in public outdoor places^1^	1.194 (2.28)	0.966 (2.24)	1.108 (2.20)	0.712 (1.55)
(…) at parties or celebrations^1^	1.543 (2.57)	1.114 (2.22)	1.481 (2.38)	1.094 (2.02)
(…) at home or someone else’s home^1^	0.960 (2.07)	0.615 (1.44)	1.104 (2.28)	0.727 (1.66)
Glasses of alcohol consumed in outdoor public places^1^	1.640 (2.14)	1.26 (2.056)	1.478 (2.16)	1.273 (2.20)
(…) at parties or celebrations^1^	2.492 82.68)	1.980 (2.33)	2.518 (2.61)	2.05 (2.61)
(…) at home or someone else’s home^1^	1.325 (1.98)	0.983 (1.83)	1.467 (2.11)	1.358 (2.22)**
Alcohol use on last weekend^2^	0.235 (0.42)	0.112 (0.32)	0.216 (0.41)	0.113 (0.32)
Mother consumes alcohol moderately/more frequently^2^	0.233 (0.42)	0.113 (0.32)	0.281 (0.45)*	0.121 (0.33)
Father (…)^2^	0.418 (0.49)	0.181 (0.39)	0.444 (0.50)	0.192 (0.39)
Siblings (…)^2^	0.225 (0.42)	0.082 (0.28)	0.240 (0.43)	0.115 (0.32)*
Partner (…)^2^	0.179 (0.38)	0.066 (0.25)	0.152 (0.36)	0.069 (0.25)
Friends (…)^2^	0.814 (0.39)	0.342 (0.48)	0.818 (0.39)	0.398 (0.49)**
Best friend (…)^2^	0.584 (0.49)	0.249 (0.43)	0.543 (0.50)	0.248 (0.43)
Mother binge drinks moderately/more frequently^2^	0.051 (0.22)	0.027 (0.16)	0.073 (0.26)	0.022 (0.15)
Father (…)^2^	0.156 (0.36)	0.067 (0.25)	0.160 (0.37)	0.044 (0.20)*
Siblings (…)^2^	0.128 (0.33)	0.053 (0.22)	0.150 (0.36)	0.053 (0.23)
Partner (…)^2^	0.119 (0.32)	0.044 (0.21)	0.111 (0.31)	0.057 (0.23)
Friends (…)^2^	0.683 (0.47)	0.272 (0.45)	0.640 (0.48)	0.309 (0.46)
Best friend (…)^2^	0.446 (0.50)	0.190 (0.39)	0.388 (0.49)**	0.196 (0.40)
Family alcohol consumption^3^ (BD of the mother, father and/or siblings)	0.336 (0.63)	0.147 (0.47)	0.491 (0.66)	0.119 (0.39)
*Consumption of other substances*				
Number of cigarettes a week^1^	4.361 (13.92)	4.257 (16.35)	4.047 (15.07)	3.333 (12.37)
Number of shishas or hookahs a week^1^	0.821 (2.49)	0.652 (1.68)	1.050 (2.77)	1.053 (3.44)*
Smoker^2^	0.224 (0.42)	0.168 (0.38)	0.234 (0.42)	0.196 (0.40)
User of cannabis^2^	0.061 (0.24)	0.053 (0.22)	0.085 (0.28)*	0.079 (0.27)*
Prescribed tranquilizers, sedatives or sleeping pills^2^	0.019 (0.14)	0.026 (0.16)	0.032 (0.18)	0.028 (0.16)
Not prescribed tranquilizers, sedatives or sleeping pills^2^	0.012 (0.11)	0.019 (0.14)	0.028 (0.16)**	0.026 (0.16)

Note: ^1^ Continuous variable; ^2^ Dichotomous variable; ^3^ Categorical variable

In the context of engaging in binge drinking for family members, “occasionally or more frequently” is compared with “never or almost never”. Initially, these items had four response options, from “never” to “more frequently”; they were subsequently, were dichotomized.

^a^We show the average values and standard deviations in brackets. ***, ** and * represent statistically significant differences at 1, 5 and 10% between values of variables in intervention and control group (2nd and 4th column) in pre- intervention or baseline period

^b^We show the average values and standard deviations in brackets. ***, ** and * represent statistically significant differences at 1, 5 and 10% between values of variables in intervention and control group (3rd and 5th column) in post- intervention or follow-up period

### Measures and procedures

The measurements included in this study were collected by means of an online questionnaire administered during school hours. Information was collected at two different points in time [baseline (January–February) and at the 4-month follow-up (May–June)]. The questionnaire was divided into the following sections: socioeconomic variables (age, sex, family composition, nationality, economic situation at home, parents’ education level, weekly pocket money, etc.), alcohol use (binge drinking occasions in the last month, alcohol use in the last week, etc.), other substances use and social influences (parents, sibling, friend’s alcohol use, etc.) [31]. Only the number of BD occasions in the last 30 days and variables related to alcohol use were assessed at two assessment points, pre- and post-intervention. The rest of the variables were assessed only at baseline (also called the pre-intervention period). Definitions of these variables are explained below.

Within socioeconomic variables, age was calculated by dividing the difference between the date on which the subject completed the pre-intervention questionnaire and the subject’s birth date by 365.25.

Family composition was determined by means of the question “What is the composition of your family?”, with multiple answers being allowed from the following response options: mother, father, brother(s)/sister(s) who live(s) at home, brother(s) who do/do(es) not live at home, sister(s) who do/do(es) not live at home, other (nominal response). We created a new categorical variable called “family composition”, which classified families as “nuclear”, “extended nuclear”, “divorced”, “extended divorced”, “reconstituted” and “other”, but due to the large proportion of nuclear families in the sample, we dichotomized this into “nuclear” and “others”.

Nationality was a dichotomous variable with two response options: 1 if the respondent was Spanish and 0 if the respondent had another nationality or nationalities. When adolescents answered “other(s)”, they specified the additional nationality(ies). Given the proportion of responses for each other nationality, this variable was used in the analyses only as a dichotomous variable.

Religion was determined through a categorical variable with several answers, which were recoded in three response options: “Catholic”, “other religion”, “no religion”.

Parents’ educational level was calculated according to years of schooling. Initially, we collected data on the educational level with a categorical variable by asking “What is the highest level of education achieved by your father/mother?”

The economic situation at home was obtained using the question “Of the following situations, which one would you identify with the most?”. The response options were: (a) We have many economic problems at home and we don’t make it to the end of the month; (b) We manage economically at home, but we have trouble making it to the end of the month; (c) We are pretty well off economically and we make it to the end of the month; and (d) We are very well off economically. This variable was converted into a dummy variable, with a value of 1 indicating “good economic situation at home” [including options (c) and (d)], and a value of 0 indicating “other economic situation” [including options (a) and (b)]. This question was developed ad hoc and used in another study carried out by Lima-Serrano et al. [32].

To measure weekly pocket money, we asked about the amount of money the subject had available to spend on his/her appearance weekly (not including money for clothing or his/her savings). Response options were: “0 euros”, “up to 10 euros”, “between 11 and 20 euros”, “between 21 and 30 euros”, “more than 30 euros”. Due to the proportions of responses in each category, these amounts were recoded into three categories: €0, between €1 and €20, and more than €20. Similar recoding was used in a study carried out by Díaz-Geada et al. [33] This categorical variable was converted to a continuous variable using the mean number of euros for each option.

Family functionality was measured using the family APGAR questionnaire [34–37], which is a tool frequently utilized in primary care and general medicine settings to assess family function through a five-item questionnaire measuring five constructs (adaptability, partnership, growth, affection and resolve).

In relation to alcohol use, the model’s endogenous variable was the number of BD occasions in the last 30 days, obtained directly from the answers given by the adolescents to the question: “During the last month, how many times did you drink 4 glasses or more of alcohol (if you are a girl) or 5 glasses or more of alcohol (if you are a boy) on one single occasion (e.g., in a bar, at a party, etc.)?”. The word “glass” was used in the question to refer to a standard drink, and an image was provided in the questionnaire to help respondents understand the meaning of a standard beverage unit or glass of alcohol. The definition of binge drinking used in this study is consistent with ESTUDES [15], as well as with the definition used by Jander et al. [38], as *Alerta Alcohol* is an adaptation of the Dutch programme Alcohol Alert [30]. One alcoholic drink equivalent in the Dutch programme was defined as a drink containing 9.9 g of pure alcohol, which is similar to the measure in Spain, where one standard unit of alcohol is equivalent to 10 g of alcohol. This variable was self-reported in both pre-intervention and post-intervention periods. The units of this measure were event counts.

With regard to other substance use, tobacco use was a categorical variable (with nine response options from “never smoke” to “daily”), which was converted to a dummy variable, with a value of 1 for “smoker” and a value of 0 for “non-smoker”. Those who reported being smokers were asked about the number of cigarettes and shishas they consumed in terms of a numerical variable.

Regarding social influences, family alcohol consumption was calculated on the basis of binge drinking among family members, i.e., the mother, the father and siblings. Three items were taken from the questionnaire that asked about the frequency of those family members who consumed 4–5 glasses of alcohol or more on a single occasion (Response options: “never”, “almost never”, “occasionally”, “more frequently”). Subsequently those items were dichotomized (1: mother/father or siblings occasionally or more frequently consumed 4–5 glasses of alcohol or more on a single occasion; 0: mother/father or siblings never or almost never consumed 4–5 glasses of alcohol or more on a single occasion). Then, the items were combined in a single ordinal variable with four response options (0: mother, father and siblings did not engage in binge drinking; 1: mother or father or siblings engaged in binge drinking; 2: two members of the family (mother, father or siblings) engaged in binge drinking; 3: mother, father and siblings engaged in binge drinking).

Additional variables were created in order to carry out the analysis. The latter included variables that indicated how close in time the subject was to the most popular local events in each city (there were a large number of events during the study period) and how much time had elapsed since the last weekend when the subject completed the questionnaire. The first variable was taken into account because binge drinking is a pattern of alcohol consumption that usually occurs during weekends and summer and spring vacations, on holidays (e.g., New Year’s Eve) and at parties such as graduation events, and at sporting events [39]. To obtain the first variable, we codified the date on which the questionnaire was completed and the date of the closest local event for each city and then calculated the difference between the two codes. For the second variable, we recoded the date on which the subject completed the questionnaire as the corresponding day of the week. The variable was then dichotomized into subjects who completed the questionnaire on Monday or Tuesday and subjects who completed the questionnaire later in the week. In relation to the first session, all participants were asked to answer the initial questionnaire for the same event, which was Christmas.

### Data analysis

We used panel count data for the empirical analysis. The analysis to determine the factors associated with binge drinking in the sample studied was conducted using three econometric procedures: a negative binomial, a finite mixture model and a two-part model. Given the nature of the endogenous variable (count data), we used a negative binomial specification, which would resolve the main drawback of the Poisson model in which the variance is equal to the mean. The data showed greater variance than average due to multiple causes, notably the high frequency of zeros.

The basic idea of these models (negative binomial regression models) is that the zeros (all or part of them) do not come from the same data-generating process as the rest of the values. For instance, in this study, for those who had engaged in binge drinking zero times, the reason might be because they are non-drinkers (they never drink alcohol) or are alcohol users (they usually drink alcohol) but are not binge drinkers, or it might also be because, even though they are binge drinkers, they had not engaged in binge drinking in the last month. In the baseline period, 33.28% of adolescents were non-drinkers and 66.72% were alcohol users. Of those who were alcohol users, 57.8% were binge drinkers. In the total sample (non-drinkers and alcohol users), 38.6% were binge drinkers.

The two-part model was used, along with the finite mixture model, to explain the probability of not reporting binge drinking and how often it happened, given that the two variables could be completely independent of each other. Finite mixture models have received increased attention in recent years due to their usefulness for modelling heterogeneous data with a finite number of unobserved sub-population and the probability of belonging to each unobserved group in order to estimate distinct parameters of a regression model or distribution in each group, to classify individuals into the groups and to draw inferences about how each group behaves [40].

The aim in using various models other than the Poisson model was to allow for greater flexibility, bearing in mind the main disadvantage of the Poisson model, which is the difficultly in capturing overdispersion – i.e., when the conditional variance exceeds the conditional average. Specifically, the first part of the two-part model is estimated using a logit regression model and the second part is specified as a generalized linear model panel regression. This model was used because of the presence of a large proportion of zero count observations [41]. In the dataset, the number of binge drinking occasions was zero for 60.95% pre-test and 67.32% post-test.

Additionally, the introducing interactions between socioeconomic variables and the intervention were estimated. However, none of these interactions turned out to be statistically significant, so we decided to use the entire sample for this analysis.

The analysis was conducted using Stata (version 16.0; StataCorp, College Station, TX, USA).

### Ethics approval

The study received approval from the Bioethics Committee of Andalusia. Written informed consent was obtained from parents and students prior to participation in the study. The questionnaires were self-completed by the adolescents and confidentiality was ensured.

## Results

### Alcohol and other substance use

Regarding alcohol use, in the IG, 40.03% of adolescents had consumed 4 or 5 glasses of alcohol on a single occasion in the last month in the pre-test period; in the CG, the proportion was 36.44%. Regarding other drug use, in the IG, 22% smoked a mean of 4.36 cigarettes (SD = 13.9; 95% CI: 2.92–5.80) and a mean of 0.82 shishas per week, and 6% were cannabis users. In the CG, 23% of subjects smoked a mean of 4.04 cigarettes (SD = 15.06; 95%CI: 2.19–5.90) and a mean of 1.05 shishas per week; 8% were cannabis users.

### Dealing with missing data

Due to the high desertion rate (> 50%) in the post-intervention period, for the majority of variables in the follow-up questionnaire we decided not to use multiple imputation and instead carried out the analysis with pairwise deletion. In relation to the main differences between subjects who replied to the post-questionnaire and those who did not reply to it (missing subjects), we found that the missing subjects were older, their father’s and mother’s schooling years were lower, the current job situation of the mother was worse, they had a worse economic situation at home and they had higher weekly pocket money. Moreover, in relation to alcohol use, those who did not answer the post-intervention questionnaire had engaged in binge drinking more frequently and consumed more alcohol in the last week; they also had friends/a best friend who consumed alcohol more frequently. In addition, a higher proportion of those who did not answer the questionnaire in the post-intervention period were smokers. These differences were statistically significant. The differences between the missing and not missing subjects did not, however, affect the results of the analyses, so the entire sample was analysed.

### Social, economic and family factors associated with binge drinking

Table 2 shows the variables associated with binge drinking among adolescents.

**Table 2:** Intervention marginal effects on BD: two-part model (logit + glm)

		*Marginal effects*
*Intervention*		
	Period	0.279 (0.20)
	Treated	0.201 (0.14)
*Socioeconomic*		
	Age	0.265 (0.05)^***^
	Female	0.068 (0.09)
	Spanish	0.226 (0.25)
	Nuclear family composition	−0.110 (0.08)
	Mother’s schooling years	− 0.002 (0.00)^*^
	Good economic situation at home	0.030 (0.09)
	Pocket money (weekly)	0.022 (0.01)^***^
	Completed questionnaire more days after last weekend	− 0.330 (0.11)^***^
	Completed questionnaire near to local events	− 0.001 (0.00)
*Alcohol*		
	Family alcohol consumption	0.502 (0.06)^***^
	N x T	1638
	Wald χ^2^	182.72 (0.00)
	Log likelihood	− 1432.04
	Pseudo-R2 1st part	0.0774

Note: we show the parameter estimates and standard deviations in brackets. ^***^, ^**^ and ^*^ represent statistical significance at 1, 5 and 10%; 1000 replications were used for bootstrapping and standard errors were clustered at classroom level. In the two-part model and the finite mixture model, regressions were also controlled by period of intervention, female sex, age, Spanish nationality, having a partner, years of schooling of the mother, nuclear family, pocket money, family pressure, answering the questionnaire late in the week and answering the questionnaire near the date of local events

The two-part model showed the highest performance level by far with respect to statistical measures according to the value of the log-likelihood function, which is a method that represents the combination of model parameter values that maximize the probability of drawing the sample obtained. In this model, age, weekly pocket money, mother’s years of schooling, the variable “completed the questionnaire more days after last weekend” and family alcohol consumption continue to be statistically significant. There was a positive association between age, weekly pocket money, family alcohol consumption and the number of occasions of binge drinking and a negative association between the mother’s years of schooling and the variable “completed the questionnaire more days after last weekend”. On the one hand, in relation to variables that showed a positive association, the average of BD occasions increased 0.265 times per additional year, 0.022 times for weekly pocket money and 0.502 times as frequency of BD increased in the family. On the other hand, with regard to those variables showing a negative association, the average of BD occasions decreased 0.002 times for every additional year of schooling for the adolescent’s mother and decreased 0.33 times for every additional day elapsed since the last weekend for the adolescent completing the questionnaire. In terms of how big these associations are, only age and family alcohol consumption showed a relatively high value: 0.14 and 0.26, respectively, times the standard deviation for the control group at baseline period.

## Discussion

This study analysed socioeconomic and family factors associated with binge drinking in adolescents between 15 and 19 years of age enrolled in public high schools. The related variables, according to the model that showed the highest performance level in terms of statistical measures, were age, pocket money, questionnaire completed more days after last weekend and family alcohol consumption. Several studies have explored the relationship between socioeconomic variables and drinking behaviour in adolescence through cross-sectional studies [42, 43], although the use of longitudinal studies on substance use is growing. These studies have the potential to contribute to prevention science on how best to reduce problem substance use. Importantly, our findings extend research in this field by analysing and quantifying an association between socioeconomic factors and behavioural patterns of binge drinking in adolescence.

Regarding age, our findings are consistent with those of other studies [33, 44–46] that show that alcohol use, as well as the use of other drugs, increases with age. Moreover, this study showed an association between availability of money and excessive alcohol use, finding that as weekly pocket money increases, so does the likelihood of consuming alcohol. Bosque-Prous et al. [47] reported similar findings, such as that high weekly student income was associated with a higher likelihood of alcohol consumption for different drinking measures (weekly binge drinking and weekly alcohol consumption). This finding is important for informing families that a greater availability of money could contribute to this risk behaviour.

In relation to completion of the questionnaire more days after the last weekend and the association of this variable with lower drinking, it might be assumed that memory could explain this finding and, as a consequence, that the reported data related to binge drinking frequency would be underestimated, since BD is an alcohol consumption pattern that usually occurs during weekends [46].

Family alcohol consumption, as mentioned above, was positively and significantly associated with the number of occasions of binge drinking in the last month. Along the same lines, Pedersen and von Soest [48] found that binge drinking among parents was predictive of binge drinking among their children, just as the frequency of alcohol use by parents is predictive of the frequency of alcohol use by their children. Other authors, such as Moore et al. [49], affirm that parents’ behaviours are central influences on adolescent activities and, specifically, on alcohol use.

No consistent and significant associations were found in our study between perceived economic situation at home and binge drinking. However, Liu et al. [23] showed that girls from high perceived family wealth groups were more likely to be abstainers than girls from low perceived family wealth groups. Conversely, ESTUDES (2016–2017) [16] found an association between higher family socioeconomic level and higher alcohol use among adolescents. In addition, Moure-Rodríguez et al. [50] found that a high maternal education level – a variable considered reflective of a high socioeconomic level – was a risk factor for risky consumption (drinking 5 alcoholic beverages or more for girls and 6 or more for boys) among Spanish youth. However, they did not find an association between maternal education level and heavy episodic drinking (frequency of drinking 6 or more alcoholic beverages per occasion). On the one hand, this inconsistent association could be related to the positive perception (normalization) of alcohol consumption in Spanish culture and the easy accessibility of alcohol, independently of economic status. On the other hand, it could be argued that, there is a high tendency towards street drinking among Spanish adolescents, especially in Andalusia, Spain, where they can get alcoholic drinks in stores at a lower price than in other regions.

Regarding gender, this study found that the number of occasions of binge drinking was higher among girls. There are various recent studies that reflect this trend towards equalizing consumption between women and men [33, 50]. Other studies have found different results using other variables. For example, Wilkinson et al. [51] measured the association between adherence to gender-typical behaviour and substance use from adolescence into young adulthood, and found that greater gender-adherence in females is associated with lower odds of high-frequency substance use. At the same time, male adolescents who are more gender-adherent, compared with less adherent males, have a higher frequency of binge drinking. The data from the Health Behaviour in School-aged Children survey (2013–2014) at the European level showed that alcohol use still tends to be more common among boys, but gender differences appear to be decreasing, particularly in relation to weekly drinking and drunkenness on more than one occasion. These differences could be related to the population and dates studied [44].

Spanish students reported higher alcohol consumption than non-Spanish students. This finding is similar to a study carried out in the United States [52], which showed that US-born adolescents reported higher use. It seems that living in one’s birth country is related to alcohol use in the Spanish context. In their study, Díaz Geada et al. [33] found that being a non-immigrant increases the probability of buying alcohol among adolescents between 14 and 18 years of age. This fact could be associated with a higher socioeconomic level or greater availability of economic resources, as well as with the ease of access or acquisition of alcoholic beverages for natives [33]. Moreover, it has been known for many years that alcohol is an integral part of the cultural and culinary heritage of western European countries, particularly those that border the Mediterranean Sea [53, 54].

One of the main limitations of our study might be missing data from the post-test, which was mainly related to the early completion of classes by vocational training students (whose classes ended before those of the other participants). In addition, the date for administering the post-intervention or follow-up questionnaire fell close to the final examination period in the schools involved, which made it difficult to ensure that the questionnaire was completed at school. The high dropout rate prevented us from using multiple imputation. High attrition rates, which are known to be common in eHealth interventions, may have affected the outcomes of the analysis [55, 56]. This attrition rate limited us to using only the variable “economic situation at home” as a mirror of family socioeconomic status, owing to the loss of data from a question about the parents’ occupation. Another limitation is that the questionnaire was self-reported. This could have caused students to respond according to what they considered socially acceptable, although confidentiality was ensured during the completion of the questionnaire. The upside of using data collected from self-reported measures is that, if answers had been given by other people, such as parents, they might have been estimates that bore little resemblance to the reality, since parents’ perceptions of their children’s behaviour and substance use can be very different from those reported by the children themselves, as shown by Jander et al. [38]. The association between binge drinking and completing the questionnaire more days after the last weekend could be another limitation, as the passage of more time could have resulted in recall bias as to the amount consumed, since adolescents drink more frequently on weekends. Another limitation could be the non-collection of information about the type of alcoholic beverage consumed; other authors have found differences in beverage type associated with sex and family affluence, among other factors [57]. Finally, it is important to note the use of the definition of binge drinking and its equivalence in grams of alcohol per standard beverage unit as a limitation, given the difference between the grams of alcohol contained in a standard beverage in the USA and Spain.

Future research should include families of adolescents to explore other factors that might influence adolescent binge drinking, as well as other socioeconomic variables that might make it possible to measure the influence of socioeconomic status in the acquisition of this pattern of consumption.

## Conclusions

In conclusion, the results of this study could complement prevention policies by emphasizing the importance of reducing weekly pocket money or the general availability of money to adolescents and highlighting the influence of binge drinking by parents and siblings and the need for the inclusion of families in interventions aimed at preventing alcohol use in adolescence. Given that family members’ alcohol use predicts binge drinking in adolescents, future studies may need to consider interventions aimed at preventing alcohol use in family members, too. In addition, given the findings relating to age, future research aimed at intervening in early adolescence to prevent binge drinking would be justified.

## Data Availability

The datasets used and/or analysed during the current study are available from the corresponding author on reasonable request.

## Abbreviations

BD: Binge drinking
IG: Intervention group
CG: Control group
WHO: World Health Organization
DALYs: Disability-adjusted life years
ESPAD: European School Survey Project on Alcohol and Drugs
ESTUDES: Spanish Survey on Drug Use in Secondary Schools
SD: Standard Deviation
